# Mindfulness training reduces slippery slope effects in moral decision-making and moral judgment

**DOI:** 10.1038/s41598-023-29614-9

**Published:** 2023-02-20

**Authors:** Wei Du, Hongbo Yu, Xinghua Liu, Xiaolin Zhou

**Affiliations:** 1 School of Psychological and Cognitive Sciences and Beijing Key Laboratory of Behavior and Mental Health, Peking University, Beijing, 100871 China; 2 Department of Psychological and Brain Sciences, University of California Santa Barbara, Santa Barbara, CA 93106 USA; 3 Shanghai Key Laboratory of Mental Health and Psychological Crisis Intervention and School of Psychology and Cognitive Science, East China Normal University, Shanghai, 200062 China; 4 PKU-IDG/McGovern Institute for Brain Research, Peking University, Beijing, 100871 China

**Keywords:** Human behaviour, Social behaviour

## Abstract

Extant research has demonstrated the positive *intrapersonal* effects of mindfulness training. However, the cognitive mechanisms underlying the effects of mindfulness training on *interpersonal* processes are less clear. Here, we combined a randomized control mindfulness training design with computational approach to moral decision-making and moral judgments. Participants were randomly assigned to a Training group (N = 32) who received an 8-week mindfulness training or a Control group (N = 26) who waited for the same period of time. Before and after the 8-week period, participants completed a moral decision-making task, where they made tradeoff between money for themselves and unpleasant electric shocks to another person, and a moral judgment task, where they evaluated the blameworthiness of someone else’s choices in the same moral decision-making task. Trait mindfulness, as measured by the Five-Facet Mindfulness Questionnaire, significantly increased from the pre- to post-training session for the Training group, but not the Control group, demonstrating the effectiveness of the mindfulness manipulation. For the Control group, participants’ moral preference in both the decision-making task and the judgment task declined over time, exhibiting a “slippery slope” effect. In contrast, for the Training group, mindfulness training prevented moral preferences from declining. Computational modeling revealed that mindfulness training specifically reduced the increase in the weights of money over time in both the decision-making and judgment tasks, thereby curbing the “slippery slope” effects. These findings provide a cognitive account of the prosocial effects of mindfulness training on moral decision-making and moral judgments.

## Introduction

Mindfulness is conceptualized as a state or trait of open and nonjudgmental awareness of and attention to one’s experiences (e.g., sensation, cognition, and emotion) in the present moment^1^. Due to its potential positive intrapersonal (e.g., better concentration and mental and physical health) and interpersonal (e.g., prosociality) outcomes, various training programs have been developed to help individuals cultivate their trait and state mindfulness. Since the early 2000s, the psychological mechanisms underlying the outcomes of mindfulness training have gained traction in psychological research. In the context of quantitative, psychological research, a standard and perhaps mostly studied operationalization of mindfulness training practice is the mindfulness-based stress reduction program (MBSR^2^)—an intervention procedure introduced as an alternative treatment for individuals with compromised physical and/or mental health^3,4^. A large body of research on mindfulness has shown that such practice has positive *intrapersonal* outcomes on the individuals, such as reducing stress and other negative emotions^5–8^, boosting positive emotions and subjective well-being^9^ (for recent meta-analysis, see^10,11^), and improving attentional control^12^. However, the *interpersonal* and moral outcomes of mindfulness training have only been a focus of social and positive psychology research^13–15^ in recent years. For example, some studies demonstrate that mindfulness increases empathy and helping behaviors^16–18^ and decreases ostracism and intergroup biases^19,20^. Note that by distinguishing the intrapersonal outcomes and interpersonal outcomes of mindfulness training, we did not mean that these two types of outcomes are necessarily driven by non-overlapping cognitive mechanisms. In contrast, it is possible that the same cognitive mechanisms underlie both intrapersonal and interpersonal domains. This conjecture is in line with theoretical work highlighting the commonalities of social and non-social processes in terms of underlying neurocognitive mechanisms^21^.

One limitation of these studies, as a recent review points out^18^, is that most of them adopted a correlational design, examining the correlation between trait mindfulness and prosocial behavior or its underlying psychological antecedents (e.g., empathy); few of them adopted a longitudinal and randomized controlled trial design. Therefore, these studies are not conclusive about the direction and causal role of mindfulness training on interpersonal and moral outcomes^4^. People’s behaviors tend to be more unethical over time, a phenomenon known as ‘moral slippery slope’^22–24^. In a previous laboratory study of this phenomenon, Garrett and colleagues^22^ used a multi-round dishonesty task to measure the escalation of participants self-serving dishonest behaviors across trials. They found that when dishonest choices were beneficial to the participants themselves (even at the cost of harming another), the degree of dishonesty escalated from trial to trial. Importantly, such escalation tendency was significantly more pronounced when the self-beneficial choices harmed another (i.e., immoral) than when such choices helped another (i.e., morally neutral or even positive). In this previous study, the “moral slippery slope” effect was defined and measured on a trial-by-trial level. However, this does not mean this phenomenon cannot exhibit itself in a longer timeframe, especially in real-world social contexts^23,24^. Such a trend seems unique to moral preference, as compared with attentional control or emotions, which renders a randomized controlled design necessary.

Another limitation of these previous studies is that they have been focused on (hypothetical) behavioral outcomes, which are the end-point products of underlying cognitive processes^25^. It is still unclear which of the underlying cognitive processes are altered by mindfulness training. Fundamental to moral cognition are trade-offs between selfish interests and harm to other individuals, groups, or society in general^26^. Such trade-offs underlie both individuals’ *decisions* about what to do and *judgments* about how moral or immoral an action is, two basic modalities of moral cognition^27^. Does mindfulness practice modulate the cognitive processes of selfish interests, harm to others, or both? Is the effect similar for moral decision-making and moral judgment?

To probe and quantitatively characterize the cognitive processes underlying moral decision-making and moral judgment, we adopted a harm aversion moral decision-making task^28^ and a harm aversion moral judgment task^29^. In this framework, harm is core to the concept of morality. However, we did not argue that harm is the only morally relevant domain. We note that both the pluralist and the harm-centered moral theories acknowledge the importance of harm in the concept of morality^26,30^. In the decision-making task, participants (i.e., Decider) made trade-offs between profiting themselves and inflicting pain on a stranger (i.e., Receiver) or themselves. Their behavioral preference in the moral decision-making task (i.e., moral preference) is characterized as the extent to which they forgo profits to reduce the pain (i.e., being harm averse) for the Receiver relative to the pain for themselves. Participants also judged the blameworthiness of others’ choices in the same setting (i.e., moral judgment). The two cognitive mechanisms involved in moral decision-making and moral judgments are the processing of one’s own material profits (i.e., money) and the harm to another person (relative to the self). Previous studies have shown that, from the Decider’s perspective, the neurocognitive processing of material profits drives the decision to maximize the Decider’s self-interest, while the neurocognitive processing of harm to another person (relative to oneself) drives the decision to minimize harming others^31,32^. From a third-party evaluator’s perspective, these two cognitive processes also influence the blameworthiness they assign to a given choice—everything else being equal, a choice that leads to more harm to another is judged as more blameworthy, whereas a choice that results in more profits to the Decider is judged as less blameworthy^28,29,33^. Because of these tasks allow researchers to quantify these cognitive processes, they have been applied to address a number of critical questions in moral psychology (for a review, see^27^), such as the neural basis of prosocial behaviors^28,34^, the cognitive mechanisms underlying moral impression updating^35^, and moral influence^32^.

Previous studies on mindfulness have identified two possible ways in which mindfulness training could influence the aforementioned cognitive processes underlying moral decision-making and moral judgment. First, mindfulness training could lead to higher empathic concern for others’ suffering^13,36^, which could then increase the weight of Receiver’s pain in decision-making and judgment. Second, mindfulness training could also make people focus more on the present moment, and reduce the motivation to seek external, material goods^1,16,37–39^. Therefore, it may reduce the weight of monetary profit in decision-making and judgment. To pinpoint these cognitive processes underlying moral decision-making, we modeled participants’ choices using drift diffusion models (DDM)^40,41^. In DDM, hypothetical evidence favoring one option over the other accumulates over time, until it crosses a boundary or threshold corresponding to an option, at which time that option is chosen. The hypothetical evidence integrates information over multiple choice attributes. In other words, the evidence accumulation speed (i.e., drift rate) scales with the magnitude of choice attributes. Therefore, the weight of a choice attribute on drift rate reflects the importance of that attribute in the decision-making process. In our moral decision-making task, one option is always more financially beneficial to the participant at the cost of more harm to the participants themselves or the Receiver, whereas the other option is always less financially beneficial to the participants and less harmful. Therefore, the decision attributes are the additional profit for the self and the additional pain for the self and the Receiver, with the former tending to drive the decision evidence to accumulate towards the more harmful and beneficial option and the latter tending to drive the decision evidence towards the less harmful and less beneficial option^32^. This analysis then allowed us test hypotheses regarding the cognitive processes underlying the impact of mindfulness practice on moral decision-making based on the weight of choice attributes.

Previous work has shown that moral judgment in the harm aversion setting is also driven by the two choice attributes, namely profit for the Decider and pain for the Receiver^28,29^. Specifically, when holding the profit constant, the more pain inflicted on the stranger, the more blameworthy the choice is judged. Conversely, when the pain inflicted is the same, the more profit one can make from it, the less blameworthy the choice is judged. In other word, from an observer’s perspective, the profits the Decider obtains justify the infliction of pain on the Receiver. Mirroring the hypotheses for the effect of mindfulness training on moral decision-making, two non-exclusive hypotheses can be proposed regarding the moral slippery slope effect in moral judgment and how mindfulness training may influence it. For one, it is possible that over time observers become adaptive to and care less about the harm. Thus, the same amount of harm leads to less harsh moral judgment (i.e., less blameworthy). Alternatively, the justifying effect of profit may increase over time, such that the same amount of money is able to justify more harm as the observers repeat the same judgment over time. To test these hypotheses, we combined the harm aversion paradigm and computational models with an 8-week mindfulness training intervention.

## Materials and methods

### Participants

Three hundred and two participants were recruited through word-of-mouth and adverts posted on social media. Sixty-eight of them met the inclusion criteria (see below) and agreed to participate in the study. The participants were randomly assigned to a mindfulness training group (hereafter Training group, N = 35) and a waiting group (hereafter Control Group, N = 33). Three participants in the Training group did not complete the program (dropout rate = 8.6%), leaving a final sample of N = 32 (26 female, *M*_age_ = 30.1 ± 8.6 years, age range = 20–53 years, mean years of education = 16.9 ± 1.2). Seven participants in the Control group did not complete the study (dropout rate = 21.2%), leaving a final sample of N = 26 (18 female, *M*_age_ = 29.2 ± 7.0 years, age range = 22–50 years, years of schooling = 17.4 ± 1.9). All subsequent analyses were performed on the data of the remaining participants. The participants were not blind to the group assignment. The experimenters of the experimental tasks were blind to the group assignment. The study was approved by the Committee for Protecting Human and Animal Subjects of Peking University. The experiment was performed in accordance with the approved protocol. Informed consent was obtained from all participants prior to the experiment.

### Inclusion criteria

**Figure 1:**
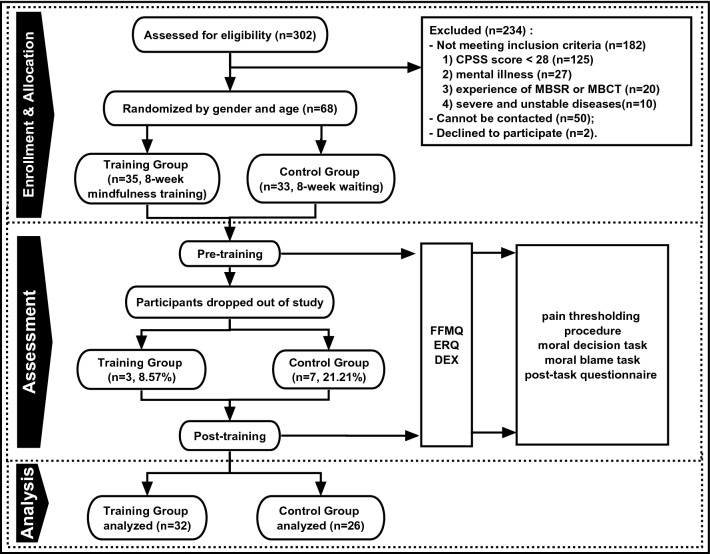
Flowchart of study design and procedure.

To be included in the study, the participants need to meet all the following requirements (Fig. 1): (a) a score of 29 or higher on the Chinese Perceived Stress Scales (CPSS^42^); (b) having no experiences of Mindfulness Based Stress Reduction (MBSR) or Mindfulness Based Cognitive Therapy (MBCT); (c) having no regular practice of yoga, meditation, or Tai-chi in the past six months (more than 20-min practice/week counts as regular practice); (d) no severe and unstable physical illnesses that may render it impossible for them to attend the trainings in-person; (e) having no psychiatric condition that met the DSM-IV-TR (American Psychiatric Association 1994) diagnosis in the past six months; (f) having no self-injury or suicidal risks, aggression or destruction behaviors; (g) a commitment of conforming to the requirements of the study as much as possible (e.g., randomization, no schedule conflicts, no attendance for other mindfulness based interventions or experiments during training).

### Mindfulness training protocol

This 8-week mindfulness training was developed based on the protocol of mindfulness-based cognitive therapy^43^, translated into Chinese by one of the authors (X. L.). Some elements of the protocol were considered inappropriate for non-clinical populations and were replaced by other meditation practices from MBSR^44^, such as yoga meditation. This program including body scanning, sitting meditation, walking meditation, yoga, and group discussions^45^, aiming to train participants to shift their attention from an internal perspective to the current state within the body, or from an external perspective to what is currently happening in a non-judgmental way. This training protocol has been validated with various Chinese populations^46–48^. The participants in the Training group received an 8-week (2.5-h per week) mindfulness training and a one-day retreat (a weekend between Week 6 and Week 7), led by two experienced instructors. These participants were also asked to practice guided meditation for 45 min daily. The intervention started in April 2019 and ended in June 2019. The participants assigned to the control group were asked to participate in positive thinking exercises after the assessment.

### Design and procedure

The study had a 2 × 2 mixed design, with a between-participant factor Group (Training vs. Control) and a within-participant factor Session (Pre- vs. Post-training). The moral decision-making task had another within-participant factor Condition (Self vs. Other; see below). The pre-training session took place a week before the 8-week mindfulness program began, and the post-training session took place a week after the program ended (Fig. 1). Participants completed trait questionnaires (Table 1) and the moral decision-making and judgment tasks once in each session.

**Table 1:** Distributions of demographic and trait variables.

	Training group (*N* = 32)		Control group (*N* = 26)	
	Pre-training	Post-training	Pre-training	Post-training
Gender	26 female		18 female	
Age	30.12 (8.61)		29.23 (7.03)	
Years of education	16.88 (1.24)		17.37 (1.90)	
Mindfulness (FFMQ)	115.13 (13.07)	132.22 (17.89)	113.69 (13.96)	113.88 (20.62)
Executive control (DEX)	51.88 (10.39)	48.72 (11.44)	54.35 (9.37)	56.42 (11.65)
Reappraisal (ERQ)	29.25 (4.33)	29.88 (3.22)	28.31 (5.28)	27.12 (5.76)
Suppression (ERQ)	13.97 (3.88)	13.75 (3.61)	13.81 (5.46)	14.85 (4.40)

#### Pain thresholding

After completing the trait questionnaires, the participant underwent a pain thresholding procedure to identify a stimulus strength that corresponds to each participant’s level-8 pain on a scale of 0 (“not painful”) to 10 (“unbearable”). This stimulus would be used in the moral decision-making task. For details of the pain thresholding procedure, please see *Supplementary Materials*.

#### The moral decision-making task

 After the pain thresholding procedure, the participant was led to a testing room and was told that they were going to complete a decision-making task with another person in a separate room. The task involved a decider role and a receiver role, which would be randomly assigned between the participant and the other person (for details of the role assignment procedure, see^49^). Unbeknownst to the participant, they would always be assigned to the Decider role. As a decider, the participant made a series of choices between a harmful option and a helpful option. The harmful option contained more monetary payoff for the participant, but delivered more electric shocks to the participants themselves in the Self condition or to the receiver in the Other condition. There were 96 trials in each condition and the trials were the same across conditions (see *Supplementary Materials* for the complete trial set). The values of the monetary payoff and the number of shocks on each trial were simulated such that across trials the money difference (Δm) and shock difference (Δs) were decorrelated (for details of the simulation, see^32^). At the end of the task, one of the participant’s choices would be randomly selected and made real.

#### The moral judgment task

In both the pre- and post-training sessions, participants completed a moral judgement task following the moral decision-making task. In this task, participants saw all of the trials in the Other condition that they just saw in the moral decision-making task. The participants’ task was to judge how blameworthy it would be if someone chose the harmful option on each trial on a visual analog scale ranging from “not at all blameworthy” to “extremely blameworthy” (see^32^).

#### Debriefing questionnaire

 At the end of each experimental session, we asked the participants to report their experience of the tasks. On a 7-point Likert scale (1 = *not at all*, 7 = *very much*), participants evaluated: (1) how unpleasant a level-8 pain was for themselves and for the Receiver; (2) how morally conflicted they felt about their decisions; (3) how blameworthy their decisions were; and (4) how guilty they felt toward the Receiver. Participants also reported how confident they were about (5) the anonymity of their choices and (6) the anonymity of their identity on a 5-point Likert scale (1 = *fully confident* to 5 = *not at all*). The two groups did not differ in any of these questions in the Pre-training session. We did not see significant changes in the ratings on the questions (2)–(6) in the Post-training relative to the Pre-training sessions. However, their reported unpleasantness for the self and for the Receiver was affected by the Mindfulness training (for details, see Table S1).

Finally, we asked participants to explain, in their own language, how they made their decisions during the experiment. No participant mentioned concerns about their reputation or reciprocity. Participants who completed the study were thanked and received 100 RMB (about 15 USD) as compensation.

### Trait measures

#### Measures of trait mindfulness

 The 39-item Chinese version^50^ of the Five-Facet Mindfulness Questionnaire (FFMQ,^51^) was used to measure trait mindfulness and its change across experimental sessions (i.e., Pre- and Post-training). Items were scored on a 5-point Likert scale with higher scores indicating more mindfulness. The scale has five subscales, corresponding to five aspects of mindfulness. In this study, the reliability of the scale was good (Cronbach’s α = 0.84), therefore we combined these sub-scales into an overall mindfulness score.

#### Emotion regulation strategies: reappraisal & suppression

 The 10-item Chinese version^52^ of the Emotion Regulation Questionnaire (ERQ, Ref.^53^) was used to assess the usage of two emotion regulation strategies, cognitive reappraisal (changing the emotion by reappraising the situation and one’s own thoughts about it; 6 items; e.g., “When I’m faced with a stressful situation, I make myself think about it in a way that helps me stay calm”) and expressive suppression (suppressing the expression of the emotion; 4 items; e.g., “I keep my emotions to myself”). Items were rated on a 7-point Likert scale ranging from “strongly disagree” to “strongly agree”, with higher scores indicating higher usage of that strategy. In the present study, Cronbach’s α was 0.73 for reappraisal and 0.74 for suppression, comparable to that in previous work using this measure^52^.

#### Executive control

 The 20-item Chinese version^54^ of the Dysexecutive Questionnaire (DEX, Ref.^55^) was used to measure everyday manifestations of dysexecutive problems. Lower frequency of dysexecutive behavior indicates higher trait executive control. Participants were asked to indicate how often they experience certain difficulties associated with control and direction of cognition, emotion, and behavior (e.g., with planning, impulsivity, motivation, etc.). Items were rated on a 5-point Likert scale ranging from “never” to “very often”, with lower scores indicating with lower scores indicating less experience of executive control failures in everyday life.

### Computational modeling of moral decision-making data

In the moral decision task, we modeled participants’ choices in the Pre- and Post-training session separately with a computational model reported in a previous study using the same moral decision-making task^28^. In this model, choices are made based on the difference in subjective value between the harmful and helpful options. The subjective value difference (ΔV) is a linear function of money difference (Δm) and shock difference (Δs) between the two options, scaled by a harm aversion parameter, κ,

$$\Delta V=\left(1-\kappa \right)\Delta m- \kappa \Delta s$$

$$\kappa =\left\{\begin{array}{*{20}l}{\kappa }_{\text{self}}, {\text{if}} \; {\text{Self}}\; {\text{trial}}\\ {\kappa }_{\text{other}},\, {\text{if}} \; {\text{Other}} \; {\text{trial}}\end{array}\right.$$

The harm aversion parameter (κ) can be understood as the exchange rate between Δm and Δs. When κ is close to 1, the Decider becomes extremely harm averse, which means they will refuse to increase the number of shocks even when the extra money they can get is very large. In contrast, when κ approaches 0, the Decider is minimally harm averse and will accept any number of shocks in exchange for a small amount of money. In this model, the harm aversion parameter was allowed to vary across conditions. Estimating a separate harm aversion for the Self and the Other condition has been an established practice for this type of task, which have been replicated in a number of studies to date^28,32–34,49,56,57^. Trial-by-trial subjective value differences were transformed into choice probabilities using a softmax function^58^,

$$P\left(harm\right) = \left(\frac{1}{1+{e}^{-\gamma \Delta V}}\right)$$

where γ is a participant-specific inverse-temperature parameter that characterizes choice noisiness. This model correctly explained 86.8% of the participants’ choices (95% confidence interval (86.6–86.9%), with mean pseudo *R*^2^ = 0.549). We optimized participant-specific parameters by using the maximum likelihood estimation.

As a comparison, we estimated a model where we combined the data from both sessions in the same model and included dummy variables to indicate the pre- and post-training session (one for the Self condition and one for the Other condition). This model, however, had a higher BIC (8592) and a lower explanatory power (pseudo R^2^ = 0.527) compared with the model we described above (BIC = 8453, pseudo R^2^ = 0.549). We therefore decided to base our statistical inferences on the model described above.

### Hierarchical drift diffusion modeling

We used the Bayesian hierarchical drift diffusion model (HDDM) package to estimate trial-by-trial parametric modulations of choice attributes (Δm, Δs) and experimental manipulations on latent decision processes^41^. The drift diffusion model assumes that a hypothetical evidence signal accumulates over time towards one of two decision boundaries, which represent the two choice options. When the evidence crosses a boundary, the choice corresponding to that boundary is made. Four independent parameters describe the evidence accumulation process: the drift rate (v) reflects the speed at which the evidence accumulates towards one option over the other; the decision threshold (a) indicates the separation between the two boundaries that the evidence needs to reach for a decision to be made; the initial bias (z) determines the starting point of the evidence accumulation process in the absence of any information about the choice attributes; and the non-decision time (NDT) quantifies the portion of reaction times that are not attributable to the evidence accumulation process, such as perception and motor response execution. Model parameters were estimated by taking into account both reaction times and choices of the observed decision data. The Bayesian framework assumes that model parameters of individual participants are random samples drawn from group-level distributions. Data from the two groups were modeled separately, and within each group, the Self and Other conditions were modeled in separate models. Altogether, four models were estimated. For each of these models, drift rate was modulated by Δm, Δs, and their interactions with experimental session. Decision threshold and initial bias were allowed to vary across experimental sessions. NDT was not modulated by choice attributes or experimental manipulations. See *Supplementary Materials* for the details of model fitting.

## Results

### Demographics and trait measures

**Figure 2:**
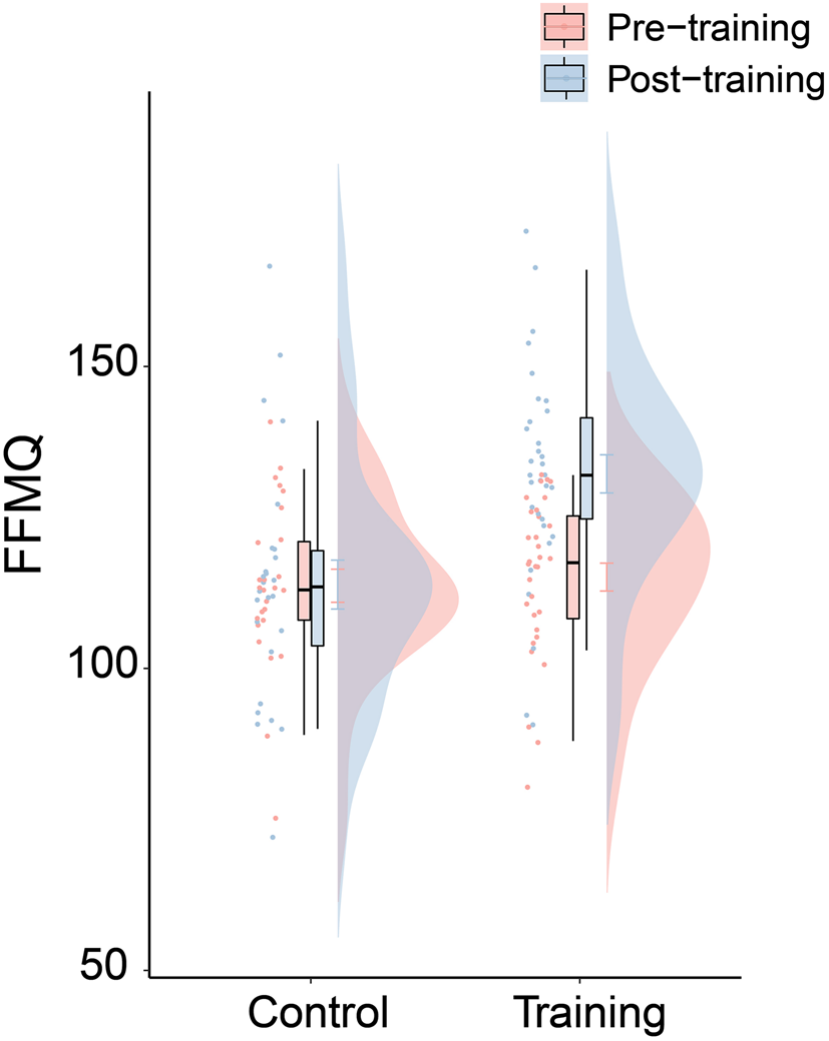
Trait mindfulness (FFMQ) depicted as a function of Group and Experimental Session. Error bars on the distribution plots display standard errors of the means. Boxes display the interquartile range (IQR) between the 25th and 75th percentile. Horizontal lines inside the boxes indicate the median.

The Training group and the Control group did not differ in terms of age (*t*(56) = 0.43, *p* = 0.671), gender (*χ*^2^ (1) = 0.57*, p* = 0.450), or years of education (*t*(56) = 1.19, *p* = 0.241). An analysis of the FFMQ score suggested that the 8-week mindfulness training significantly increased Training group participants’ trait mindfulness, while the trait mindfulness of the Control group did not change significantly (Fig. 2, Table 1). Specifically, we estimated a mixed linear effects model where we included the participants’ FFMQ score as the dependent variable, Group, Session, and their interaction as the fixed effect predictors, and participants’ age, gender, and years of education as control variables. Participant ID was included as random intercept. We found that the interaction term was significant (*B* = 16.9 ± 4.3, 95% CI = [8.5, 25.3], *b* = 0.93, *t* = 3.96, *p* < 0.001). Specifically, the between-group difference in FFMQ was not significant in the pre-training session (*B* = 2.3 ± 4.3, 95% CI = [− 6.2, 10.8], *b* = 0.13, *t* = 0.52, *p* = 0.606), but was significant in the post-training session (*B* = 19.2 ± 4.4, 95% CI = [10.7, 27.7], *b* = 1.05, *t* = 4.33, *p* < 0.001). Looking at the interaction from a different angle, for the Control group, the FFMQ did not change significantly across the sessions (*B* = 0.2 ± 3.2, 95% CI = [− 6.0, 6.4], *b* = 0.01, *t* = 0.06, *p* = 0.952); for the Training group, the 8-week MBSR training significantly increased their trait mindfulness (*B* = 17.1 ± 2.9, 95% CI = [11.5, 22.7], *b* = 0.94, *t* = 5.98, *p* < 0.001).

We estimated a similar model with DEX as the dependent variable (Table 1). Here, the interaction term was significant (*B* = − 5.2 ± 2.3, 95% CI = [− 9.7, − 0.8], *b* = − 0.48, *t* = 2.30, *p* = 0.026). Specifically, the between-group difference in DEX was not significant in the pre-training session (*B* = − 2.8 ± 2.9, 95% CI = [− 8.3, 2.7], *b* = − 0.26, *t* = − 0.98, *p* = 0.329), but was significant in the post-training session (*B* = − 8.07 ± 4.4, 95% CI = [10.7, 27.7], *b* = 1.05, *t* = 4.33, *p* < 0.001). Looking at the interaction from a different angle, for the Control group, the DEX did not change significantly across the sessions (*B* = 2.1 ± 1.7, 95% CI = [− 1.2, 5.4], *b* = 0.19, *t* = 1.23, *p* = 0.225); for the Training group, the 8-week MBSR training significantly reduced their experiences of executive control failure (*B* = − 3.2 ± 1.5, 95% CI = [− 6.1, − 0.2], *b* = − 0.29, *t* = − 2.07, *p* = 0.043).

Neither ERQ Reappraisal nor ERQ Suppression showed this pattern of change, indicating that the mindfulness training did not significantly alter participants’ emotion regulation strategies (see Table 1 and *Supplementary Results* for details).

### Effect of mindfulness training on moral decision-making

**Figure 3:**
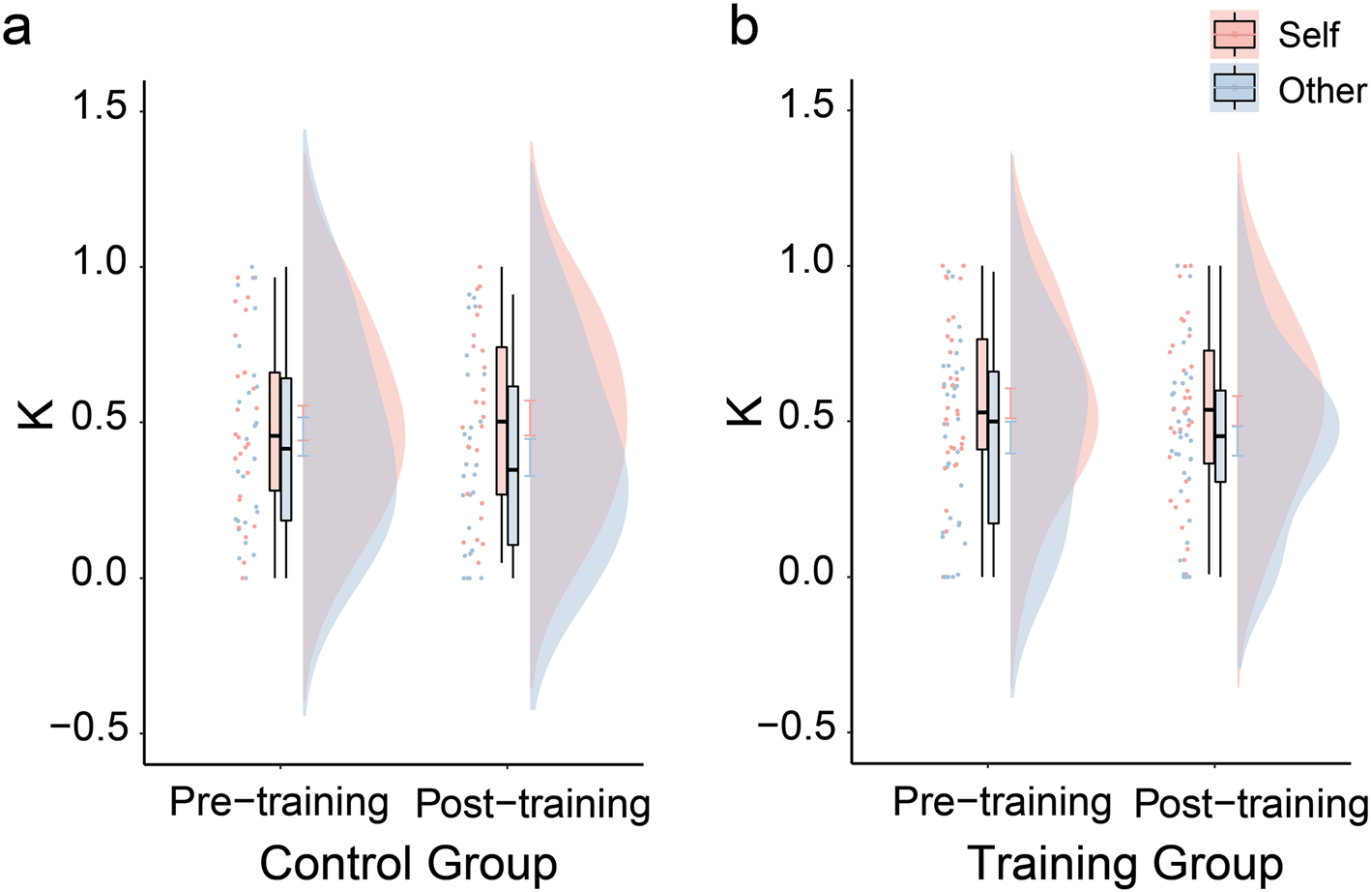
The harm aversion parameter depicted as a function of Session and Condition. (**a**) Control Group; (**b**) Training Group. Error bars on the distribution plots display standard errors of the means. Boxes display the interquartile range (IQR) between the 25th and 75th percentile. Horizontal lines inside the boxes indicate the median.

To test whether mindfulness training reduced moral slippery slope, we carried out a 2 (Condition: Self vs. Other) × 2 (Session: Pre- vs. Post-training) repeated measures ANOVA for harm aversion parameter, with Group (Training vs. Control) as a between-participant factor. If our hypothesis is true, then mindfulness training should selectively suppress the reduction in κ_other_ relative to κ_self_, in the post-training as compared with the pre-training session. Confirming our prediction, the three way interaction was significant (*F*(1, 56) = 4.66, *p* = 0.035, partial η^2^ = 0.077). Specifically, when examining the 2 (Condition: Self vs. Other) × 2 (Session: Pre- vs. Post-training) repeated measures ANOVA for the Control group, the interaction was significant (*F*(1, 25) = 4.63, *p* = 0.041, partial η^2^ = 0.156). While there was no significant difference between κ_other_ and κ_self_ in the pre-training session (M = − 0.044 ± 0.042, *t*(25) =  − 1.05, *p* = 0.303, 95% CI = [− 0.13, 0.04], Cohen’s *d* = − 0.147), κ_other_ became significantly lower than κ_self_ in the post-training session (M = − 0.126 ± 0.060, *t*(25) = − 2.09, *p* = 0.047, 95% CI = [− 0.25, − 0.00], Cohen’s *d* = − 0.426), suggesting a reduction of moral preference in the Control group from the pre-training session to the post-training session (Fig. 3a). However, the same two way interaction for the Training group was not significant (*F*(1, 31) = 0.28, *p* = 0.601, partial η^2^ = 0.009), indicating that there was no differential changes in κ_other_ relative to κ_self_ across sessions (Fig. 3b).

### Mindfulness training suppresses the increase in the weight of money on moral decision-making

We further investigated (1) which cognitive component(s) underlying decision-making underwent changes across session that gave rise to the moral slippery slope, and (2) whether mindfulness training altered such changes. To this end, we used HDDM to decompose the cognitive component(s) underlying decision-making (i.e., money maximization, pain reduction). The two groups of participants were modeled separately. A previous study using the same moral decision-making task demonstrated that value accumulation (drift rate) in this task was driven both by relative money (∆m) and relative pain (∆s) between choice options^32^. Building on this finding, here we allowed the drift rate to be weighted by relative money (*w*_money_) and relative pain (*w*_pain_) in a trial-by-trial manner. The weights were further modulated by experimental session (pre- vs. post-training). As in the previous study^32^, decision threshold (a) and initial bias (z) were allowed to vary across experimental sessions.

**Figure 4:**
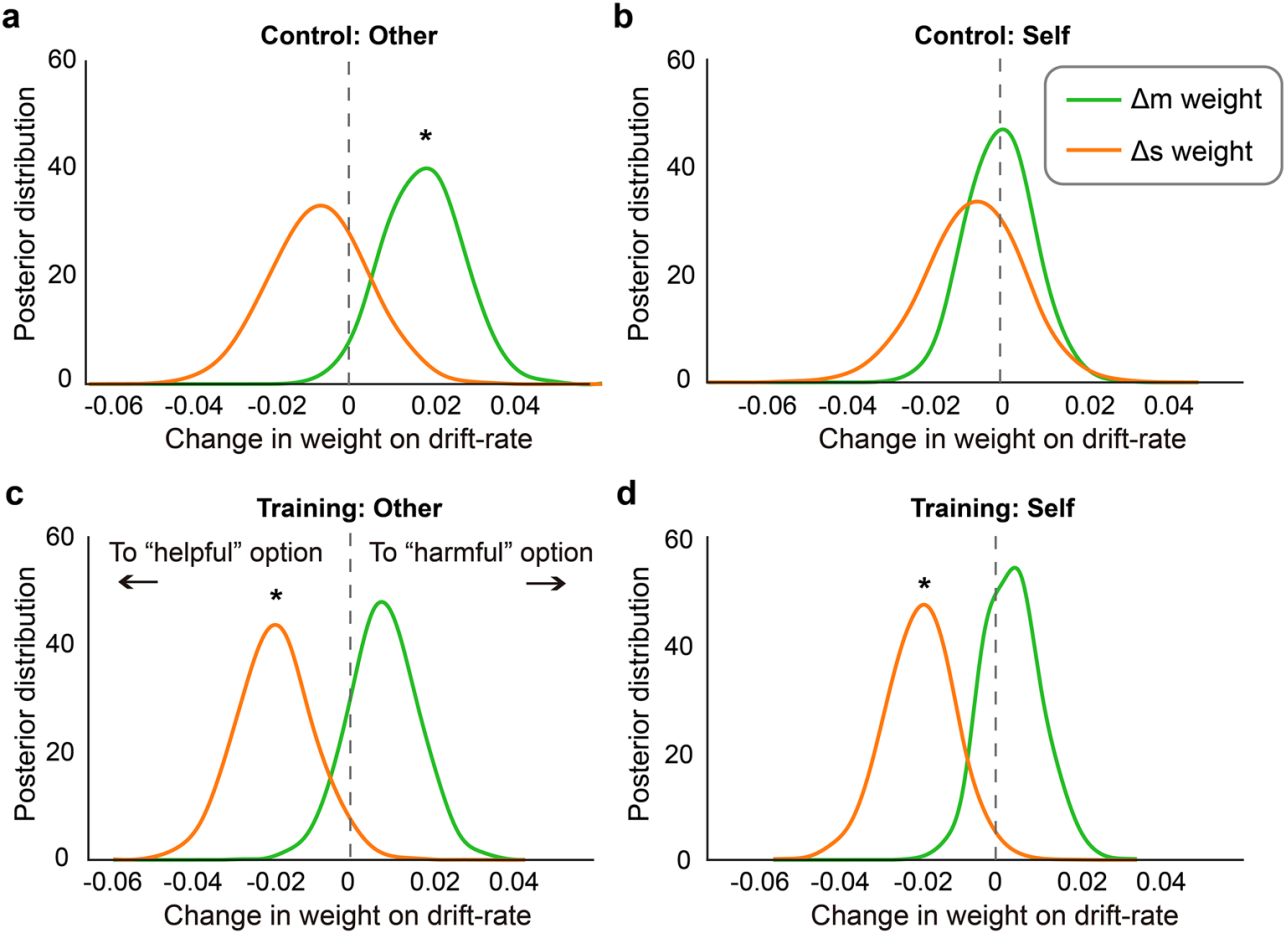
Results of drift–diffusion modeling analysis. Four separate models were estimated on the Other trials of the Control Group (**a**), the Self trials of the Control group (**b**), the Other trials of the Training Group (**c**), and the Self trials of the Training Group (**d**). For the Control group, the weight of Δm on drift rate significantly increase from the Pre- to the Post-training session in the Other condition, but not in the Self condition. For the Training group, the weight of Δm on drift rate did not change significantly between the Pre- and Post-training sessions for both the Self or the Other condition. Asterisks indicate that 0 is outside of the 95% credible interval of the distribution.

For the Control group, when the electric shocks were for the Receiver (Fig. 4a), the effect of ∆m on drift rate (i.e., *w*_money_) significantly increased in the post- relative to pre-training session (the probability of Δw_money_ being positive is 97%), indicating that obtaining additional money played a more important role in evidence accumulation towards the harmful option in the post-training session. No such change was evident for *w*_pain_ (the probability of Δw_pain_ being positive is 29%). When the shocks were for the participants themselves (Fig. 4b), neither the *w*_money_ nor *w*_pain_ underwent significant changes. For the Training group (Fig. 4c,d), *w*_money_ did not change significantly across session in both the Self and the Other conditions (the probability of Δw_money_ being positive is 59% and 81%, respectively). In contrast, *w*_pain_ became significantly more negative in both the Self and the Other conditions (the probability of Δw_pain_ being negative is 99% and 97%, respectively). Taken together, these results indicated that heightened decision weight on monetary self-interest may drive the moral slippery slope effect, and that mindfulness training prevents this effect by suppressing the increase in weight on money in moral decision-making.

### Effect of mindfulness training on moral judgment

We next investigated the manifestations of the moral slippery slope effect in moral judgment. Previous studies using the harm aversion task (only the Other condition is used in these studies) to study moral judgment have consistently demonstrated that when an observer evaluate the moral wrongness or blameworthiness of a harmful choice in the task, their judgments are strongly influenced by the two choice attributes, ∆m and ∆s. Not surprisingly, the more harm a decider causes (i.e., larger ∆s), the more blameworthy or wrong their choice is judged. ∆m, in contrast, plays an opposite role compared to ∆s, in that the more money the decider can obtain by choosing the harmful option, the less blameworthy or wrong their choice is judged, suggesting that profit to some extent justifies harmful choices^28,29^.

Here, we ran two linear mixed effect model to explain trial-by-trial moral judgment rating in terms of choice attributes (∆m and ∆s), Group (Training vs. Control), Session (Pre- vs. Post-training), and their joint effects. First, we examined whether moral judgments varied across groups and experimental sessions. In this regression, we included the main effects of group and experimental sessions, and their interaction. Participants’ harm aversion in the decision-making task (i.e., κ_other_ and κ_self_) and demographics (age, gender, and years of education) were included as covariates. Results revealed that overall participants’ judgments became more lenient in the post-training relative to the pre-training session (*B* = − 2.74 ± 1.10, CI = [− 4.88, − 0.58], *t* = 2.50, *p* = 0.013, *b* = 0.09), indicating a “slippery effect” in moral judgment. The main effect of group and the group-by-session interaction were not significant.

We next included the choice attributes (∆m and ∆s) in the regression model. We were specifically interested in whether the weights of ∆m and ∆s changed overtime and if so, whether mindfulness training had an impact on such changes. To this end, in the regression, we included the main effects of choice attributes, and all two-way and three-way interactions with Group and Session. Covariates were the same as the first model. We found that the weights of ∆m and ∆s significantly changed overtime, as indicated by significant interactions with Session (session-by-∆m: *B* = − 0.33 ± 0.15, CI = [− 0.61, − 0.04], *t* = − 2.24, *p* = 0.025, *b* = − 0.06; session-by-∆s:* B* = − 0.48 ± 0.16, CI = [− 0.79, − 0.17], *t* = -3.06, *p* = 0.002, *b* = − 0.09). Given that the main effect of ∆m was negative (i.e., reducing blameworthiness judgment) and the main effect of ∆s was positive (i.e., increasing blameworthiness judgment), the negative interactions suggested that in the post-training session the effect of ∆m on moral judgment increased while the effect of ∆s on moral judgment decreased.

Did the two groups exhibited the same degree of changes? The three-way interaction of group-by-session-by-∆s was not significant (*B* = 0.29 ± 0.21, CI = [− 0.12, 0.71], *t* = 1.39, *p* = 0.165, *b* = 0.05), indicating that the changes in the weight of ∆s did not differ across groups. In contrast, the three-way interaction of group-by-session-by-∆m was significant (*B* = 0.44 ± 0.20, CI = [0.06, 0.82], *t* = 2.26, *p* = 0.024, *b* = 0.09). Non-parametric permutation test based on Bayesian linear regression (implemented by stan_lmer in *R*) confirmed the interaction patterns (for the interaction with ∆s, CI = [0.04, 0.84]; for the interaction with ∆m, CI = [− 0.12, 0.72]).

**Figure 5:**
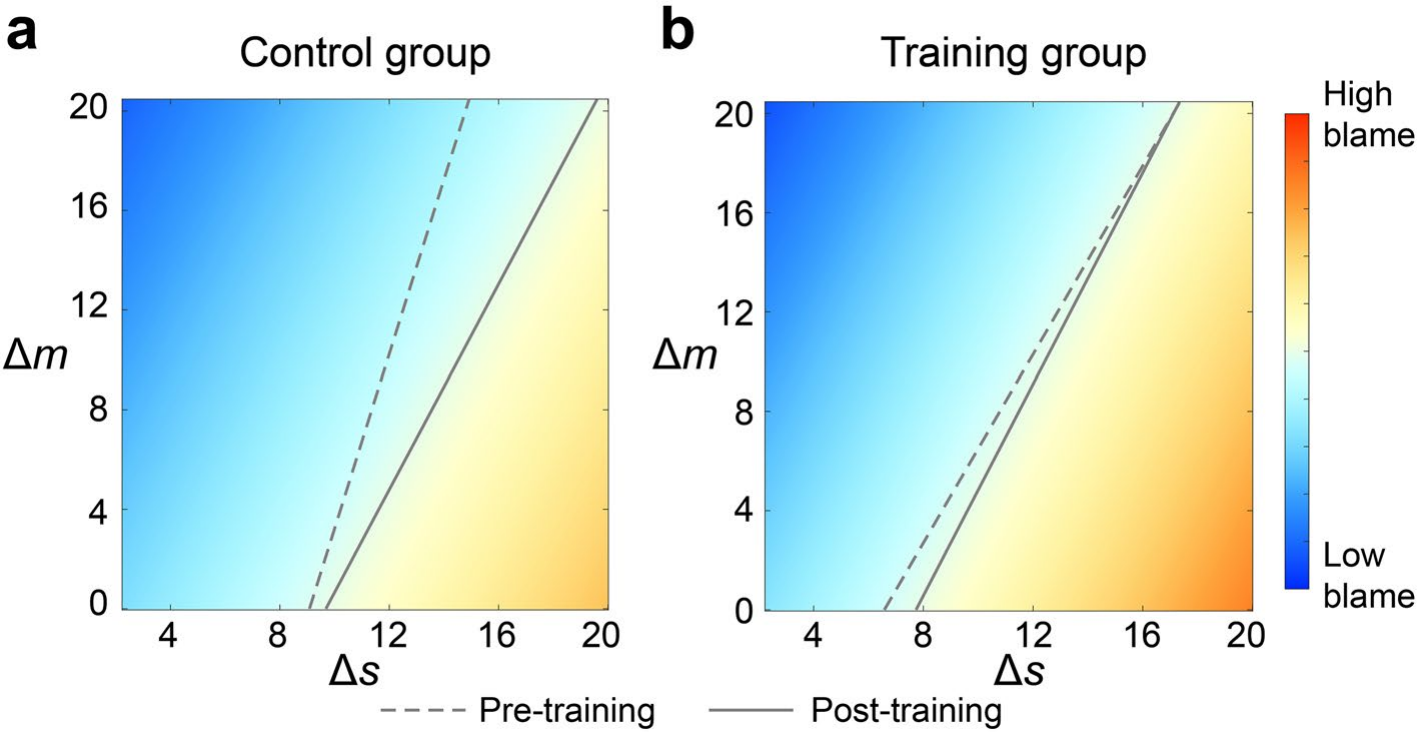
Effects of mindfulness training on the cognitive components of moral judgments. Compared with the Pre-training session (dotted line), the same amount of monetary gain (Δ*m*) justifies more harm (Δ*s*) in the Post-training session (solid line) for the Control group (**a**). This is not the case for the Training group (**b**). Color reflects blameworthiness judgment in the Post-training session.

To illustrate this, we used heatmap to display how group average blameworthiness judgment varied as a function of ∆m and ∆s, separately for each group (Fig. 5a,b). The dotted line indicates the midpoint of the blameworthiness scale (between *not at all blameworthy* and *extremely blameworthy*) in the Pre-training session, while the solid line indicates such a threshold in the Post-training session. As can be seen from Fig. 5a, the midpoint (or threshold) of blame shifted towards right in the Post-training session for the Control group, indicating that the same amount of monetary profit was able to justify more harmful consequence to the Receiver. This was not the case for the Training group (Fig. 5b). Taken together, these results indicated that people’s moral judgment on harming others for profit becomes more lenient overtime, which is primarily driven by an increase in the justifying effect of profit. Mindfulness training suppresses the increase in justifying effect of profit.

## Discussion

In the present study, we combined a randomized control mindfulness training design with computational approach to moral decision-making and moral judgments. Without any active intervention, participants’ moral preference, both in decision-making and in moral judgment, declined over time, replicating the established moral slippery slope effect^22^. Given the presence of the “moral slippery slope” effect, our mindfulness training did not positively promote or increase moral preferences relative to the baseline; instead, we found that mindfulness training prevent the moral preferences of the Training group from relapsing, as exhibited by the Control group.

One of the contributions of our study is that we provided evidence for a potential cognitive mechanism underlying the “moral slippery slope” and the potential effect of mindfulness training on it. By applying drift–diffusion modeling to the decision-making data, we showed that the decline in moral preference over time in the Control group is associated with an increase in the motivation to obtain money at the cost of harming another, rather than a decrease in the motivation to avoid harm. Importantly, the increase in such motivation was attenuated by mindfulness training, suggesting a potential cognitive mechanism underlying the prosocial effect of mindfulness training. This effect is consistent with previous findings showing that mindfulness training makes people attend to and accept the present moment, and reduces their motivation to seek external, material goods^1,16,37–39^. Our finding that it is the monetary profits component that is more sensitive to the elapse of time and mindfulness intervention is consistent with the neurocognitive basis of moral decision-making^28^. In this study, the researchers demonstrate that it is the devaluation of ill-gotten money (i.e., monetary profits obtained at the cost of harming the Receiver) in the brain valuation system that explains individual differences in the moral preference in the moral decision-making task. Our finding also echoes a recent study showing that antisocial influence, the decline of moral preference after observing another Decider whose moral preference is worse than one’s own, is primarily driven by an increase in the motivation to obtain monetary profits^32^. The fact that mindfulness training reduced training participants’ executive control failure is also in line with this argument—executive control function is positively associated with less impulse for immediate monetary reward^59,60^.

A similar cognitive mechanism may also explain the effect mindfulness on moral judgment. By separating the effects of harm and monetary profits on moral judgment (cf.^28,29^), we found that it was the monetary profits component that increased over time in the Control group. Specifically, the increase in the Decider’s motivation to obtain money via harming the Receiver may make the same choices by other Deciders less morally wrong (or more justifiable) in their eyes. For the training group, as the increase in the motivation to obtain money was attenuated, monetary gain did not become more effective in justifying harming the Receiver.

Several limitations of the present study should be noted. First, we adopted a set of strict pre-screening criteria, which led to relatively small sample sizes for both groups. Nevertheless, this sample size was equivalent to a previous study using the same moral decision-making task with a between-group design^32^. Specifically, in that study, two groups of participants (N = 34 for each group) completed the moral decision-making task once before and once after a behavioral intervention. The effect of the intervention on the harm aversion parameters for both groups were large (*r* = 0.60 and r = 0.71, respectively;^61^). Similar sample sizes are also reported in some recent studies adopting the 8-week group-based MBSR intervention procedure^62^.

The moral decision-making and judgment tasks and the computational framework behind them center around the theoretical conjecture that harm aversion constitutes utility in moral cognition. Harm aversion is a preference defined as a distaste for harming others. Admittedly, there has not been consensus regarding whether harm is the essence of morality^26,63^ or just one of several moral domains^30^. Nevertheless, it is widely acknowledged that ‘do no harm’ is a widely acknowledged moral principle^64,65^ and most frequently encountered in everyday life^66^. Future work may adapt our computational operationalization to investigate whether or not mindfulness training has similar effects on judgment and decision-making in moral domains (e.g., tradeoff between self-interest and loyalty to one’s group).

We also noted that the pre-screening criteria were independent of the moral decision-making and judgment tasks. Although such sample sizes were comparable to a recent study using the same moral decision-making task and computational analyses^32^, future work is needed to evaluate the robustness and generalizability (to different cultures and different mindfulness training programs). Second, we adopted an inactive waiting period for the Control group. To further examine the robustness of our findings, future studies adopting an active control task are needed. Third, our participants are exclusively Chinese. Although mindfulness originates from Asian religious tradition (e.g., Buddhism), the modern practice of mindfulness as a mental health intervention is more popular in Western society^37^. It is an interesting and theoretically important question to examine whether the mechanisms we identified here generalize to other cultural contexts.

To conclude, by combining a randomized control mindfulness training design with computational approach to moral decision-making and moral judgments, we demonstrate that the moral slippery slope effect is driven primarily by an increase in the consideration of self-interest over time and that mindfulness training prevents the moral slippery slope by reducing such change. These findings provide a mechanistic account of the prosocial effects of mindfulness training on moral decision-making and moral judgments.

## Supplementary Information

Supplementary Information.

## Data Availability

The datasets used and analyzed during the present study will be available from the corresponding author upon reasonable request.
