# Author Correction: Mindfulness training reduces slippery slope effects in moral decision-making and moral judgment

**DOI:** 10.1038/s41598-023-30978-1

**Published:** 2023-03-07

**Authors:** Wei Du, Hongbo Yu, Xinghua Liu, Xiaolin Zhou

**Affiliations:** 1 School of Psychological and Cognitive Sciences and Beijing Key Laboratory of Behavior and Mental Health, Peking University, Beijing, 100871 China; 2 Department of Psychological and Brain Sciences, University of California Santa Barbara, Santa Barbara, CA 93106 USA; 3 Shanghai Key Laboratory of Mental Health and Psychological Crisis Intervention and School of Psychology and Cognitive Science, East China Normal University, Shanghai, 200062 China; 4 PKU‑IDG/McGovern Institute for Brain Research, Peking University, Beijing, 100871 China

Correction to: *Scientific Reports* 10.1038/s41598-023-29614-9, published online 20 February 2023

The original version of this Article contained an error in the spelling of the author Xinghua Liu which was incorrectly given as Xinhua Liu.

The original Article has been corrected.

