# Supplementary material for: Mindfulness training reduces slippery slope effects in moral decision-making and moral judgment
Source: Sci Rep. 2023 Feb 20;13:2967. doi: 10.1038/s41598-023-29614-9 (PMC9941505; doi:10.1038/s41598-023-29614-9)
Supplement: Supplementary file 1 — Supplementary Information. [file 41598_2023_29614_MOESM1_ESM.docx]

*Supplementary Information for*

**Mindfulness training reduces slippery slope effects in**

**moral decision-making and moral judgment**

List of items included in the Supplemental Materials

1. Supplementary Methods
   1. Detailed pain thresholding procedure (p. 2)
   2. Hierarchical Drift Diffusion Modeling (p. 2)
2. Supplementary Results
   1. Results of regression analysis with ERQ (p. 3)
3. Supplementary Tables
   1. Table S1: Results of the debriefing questions (p. 4)
   2. Table S2: Posterior Predictive Checks for the Drift-diffusion Model (Training group) (pp. 5-6)
   3. Table S3: Posterior Predictive Checks for the Drift-diffusion Model (Control group) (pp. 7-8)

**Supplementary Methods**

**Detailed pain thresholding procedure**

Following a brief overview of the equipment and the pain thresholding procedure, an intra-epidermal needle electrode was attached to the back of participant’s left hand for cutaneous electrical stimulation (Inui et al., 2002) and participant-specific pain threshold was calibrated. Titration began with a low-level electric shock (0.1 mA) and the participants were asked to rate their experience of pain on scale of 0 (“not painful”) to 10 (“unbearable”). The initial rating was followed by a series of shocks, either increasing or decreasing in small increments. Self-reported experience of pain was collected after each shock until a rating of 8 was reached, which was recorded as the participant’s level-8 pain experience. The stimulation level corresponding to a subjective level-8 was used to deliver shocks based on the outcomes in the decision-making task. The pain thresholding procedure allowed us to control for heterogeneity of skin resistance and pain tolerance across participants, thus enabling us to deliver shocks of matched subjective intensity. It also provided an explicit experience of the aversive stimulus, thus allowing participants to make meaningful judgments and decisions later in the tasks.

**Hierarchical Drift Diffusion Modeling**

As in a previous study using the same moral decision-making task (Yu et al., 2021) and conforming to the standard procedure of HDDM model estimation (Wiecki et al., 2013), we used Markov chain Monte Carlo (MCMC) sampling methods to obtain the posterior distribution of parameters (generating 11,000 samples, discarding 1,000 samples as burn-in). We coded harmful choices as 1 and helpful choices as 0. Reaction times (RT) shorter than 0.3 second were excluded. Gelman-Rubin convergence statistics (R-hat) were examined to make sure the models had properly converged (Wiecki et al., 2013). Five chains were run, each with 5,000 iterations and 200 burn-in samples. No R-hat statistics were larger than 1.1, indicating good convergence (Ulrichsen et al., 2020). Posterior predictive checks were carried out to examine whether the simulated data based on the parameters derived from the models can reproduce the observed RT and choices. This analysis showed that the models satisfactorily reproduced the observed proportion of harmful decision and the means and the quantiles of RT for harmful and helpful decisions (**Tables S2, S3**). This indicates that the models could reliably reconstruct the patterns in the observed data.

**Supplementary Results**

**Results of regression analysis with ERQ**

For ERQ Reappraisal, we estimated a mixed linear effects model where we included participants’ DEX score as the dependent variable, Group, Session, and their interaction as the fixed effect predictors, and participants’ age, gender, and years of education as control variables. Participant ID was included as random intercept. The interaction term was not significant (*B* = 1.82±0.94, 95% CI = [-0.03, 3.66], *b* = 0.39, *t* = 1.93, *p* > 0.05), nor did the main effect of group (*B* = 0.98±1.23, 95% CI = [-1.35, 3.32], *b* = 0.21, *t* = 0.80, *p* > 0.05) or the main effect of session (*B* = -1.19±0.70, 95% CI = [-2.56, 0.18], *b* = -0.25, *t* = -1.70, *p* > 0.05). We run a similar model with ERQ Suppression. The interaction term was not significant (*B* = -1.26±1.05, 95% CI = [-3.30, 0.79], *b* = -0.29, *t* = -1.20, *p* > 0.05), nor did the main effect of group (*B* = 0.27±1.15, 95% CI = [-1.92, 2.45], *b* = 0.21, *t* = 0.06, *p* > 0.05) or the main effect of session (*B* = 1.04±0.78, 95% CI = [-0.48, 2.56], *b* = 0.24, *t* = 1.34, *p* > 0.05).

**Supplementary Tables**

**Table S1. Results of the debriefing questions**

|  | Training group  (*N* = 32) | |  | Control group  (*N* = 26) | | *F* test |
| --- | --- | --- | --- | --- | --- | --- |
|  | Pre-training | Post-training |  | Pre-training | Post-training |  |
| Unpleasantness_self_ | 5.41 (1.21) | 5.00 (1.24) |  | 5.08 (1.29) | 5.65 (0.94) | 11.754*** |
| Unpleasantness_other_ | 5.75 (1.22) | 5.44 (1.13) |  | 5.38 (1.02) | 5.50 (0.99) | 4.140* |
| Morally conflicted | 3.66 (1.33) | 3.66 (1.36) |  | 3.85 (1.85) | 4.00 (1.55) | 0.162 |
| Blameworthy | 3.69 (1.33) | 3.53 (1.34) |  | 3.27 (1.61) | 3.65 (1.32) | 1.651 |
| Guilty | 4.00 (1.27) | 3.72 (1.63) |  | 3.65 (1.77) | 4.00 (1.50) | 1.627 |
| Choice anonymity | 1.25 (0.76) | 1.28 (0.46) |  | 1.19 (0.49) | 1.27 (0.60) | 0.051 |
| Identity anonymity | 1.25 (0.76) | 1.22 (0.42) |  | 1.27 (0.67) | 1.42 (0.86) | 0.682 |

*Notes: F test: 2 (Group: Training vs. Control) × 2 (Session: Pre- vs. Post-training) repeated measures ANOVA, * p < 0.05, ** p < 0.01, *** p < 0.001.*

**Table S2. Posterior Predictive Checks for the Drift-diffusion Model (Training group)**

| Summary statistics | Observed | Predicted | SD of predicted | Credible | Quantile | Mahalanobis |  |
| --- | --- | --- | --- | --- | --- | --- | --- |
| **Training group: Self** |  |  |  |  |  |  |  |
| % of harmful decision | 0.47 | 0.47 | 0.25 | TRUE | 47.86 | 0.01 |  |
| Mean harmful RT | 1.99 | 2.28 | 0.80 | TRUE | 41.08 | 0.35 |  |
| 10q of harmful RT | 1.03 | 1.36 | 0.64 | TRUE | 33.98 | 0.53 |  |
| 30q of harmful RT | 0.85 | 1.12 | 0.40 | TRUE | 30.41 | 0.69 |  |
| 50q of harmful RT | 1.33 | 1.45 | 0.51 | TRUE | 43.66 | 0.23 |  |
| 70q of harmful RT | 1.81 | 1.85 | 0.67 | TRUE | 48.84 | 0.06 |  |
| 90q of harmful RT | 2.39 | 2.48 | 0.92 | TRUE | 49.40 | 0.10 |  |
|  |  |  |  |  |  |  |  |
| Mean helpful RT | 1.94 | 2.19 | 0.73 | TRUE | 60.93 | 0.34 |  |
| 10q of helpful RT | 1.02 | 1.32 | 0.61 | TRUE | 35.46 | 0.49 |  |
| 30q of helpful RT | 0.79 | 1.08 | 0.38 | TRUE | 24.26 | 0.75 |  |
| 50q of helpful RT | 1.29 | 1.39 | 0.47 | TRUE | 42.81 | 0.21 |  |
| 70q of helpful RT | 1.78 | 1.77 | 0.61 | TRUE | 51.95 | 0.00 |  |
| 90q of helpful RT | 2.28 | 2.38 | 0.84 | TRUE | 48.14 | 0.12 | |
|  |  |  |  |  |  |  | |
| **Training group: Other** |  |  |  |  |  |  | |
| % of harmful decision | 0.58 | 0.59 | 0.25 | TRUE | 50.85 | 0.06 | |
| Mean harmful RT | 1.90 | 2.24 | 0.82 | TRUE | 35.58 | 0.41 | |
| 10q of harmful RT | 1.04 | 1.31 | 0.59 | TRUE | 34.72 | 0.46 | |
| 30q of harmful RT | 0.74 | 1.11 | 0.43 | TRUE | 24.09 | 0.85 | |
| 50q of harmful RT | 1.22 | 1.44 | 0.54 | TRUE | 37.93 | 0.40 | |
| 70q of harmful RT | 1.72 | 1.84 | 0.68 | TRUE | 44.29 | 0.18 | |
| 90q of harmful RT | 2.30 | 2.45 | 0.92 | TRUE | 44.79 | 0.16 | |
|  |  |  |  |  |  |  | |
| Mean helpful RT | 2.05 | 2.19 | 0.83 | TRUE | 57.17 | 0.16 | |
| 10q of helpful RT | 1.05 | 1.29 | 0.66 | TRUE | 36.41 | 0.36 | |
| 30q of helpful RT | 0.82 | 1.09 | 0.43 | TRUE | 32.84 | 0.63 | |
| 50q of helpful RT | 1.38 | 1.39 | 0.54 | TRUE | 49.71 | 0.02 | |
| 70q of helpful RT | 1.91 | 1.78 | 0.69 | TRUE | 57.55 | 0.19 | |
| 90q of helpful RT | 2.49 | 2.39 | 0.95 | TRUE | 54.36 | 0.11 | |

*Notes: 10q ~ 90q: 10^th^ ~ 90^th^ quantile of RT distribution; Credible: whether observed data falls in the 95% credible interval of the simulated data; Mahalanobis: Mahalanobis distance of the observed data from the center of distribution of the simulated data.*

**Table S3. Posterior Predictive Checks for the Drift-diffusion Model (Control group)**

| Summary statistics | Observed | Predicted | SD of predicted | Credible | Quantile | Mahalanobis |  |
| --- | --- | --- | --- | --- | --- | --- | --- |
| **Control group: Self** |  |  |  |  |  |  |  |
| % of harmful decision | 0.52 | 0.52 | 0.25 | TRUE | 45.00 | 0.03 |  |
| Mean harmful RT | 1.93 | 2.15 | 0.60 | TRUE | 38.51 | 0.37 |  |
| 10q of harmful RT | 0.93 | 1.33 | 0.51 | TRUE | 16.95 | 0.79 |  |
| 30q of harmful RT | 0.91 | 1.04 | 0.46 | TRUE | 44.16 | 0.27 |  |
| 50q of harmful RT | 1.37 | 1.35 | 0.50 | TRUE | 57.72 | 0.04 |  |
| 70q of harmful RT | 1.75 | 1.74 | 0.56 | TRUE | 56.00 | 0.02 |  |
| 90q of harmful RT | 2.27 | 2.35 | 0.70 | TRUE | 50.07 | 0.10 |  |
|  |  |  |  |  |  |  |  |
| Mean helpful RT | 1.90 | 2.22 | 0.68 | TRUE | 65.62 | 0.46 |  |
| 10q of helpful RT | 0.98 | 1.37 | 0.52 | TRUE | 20.78 | 0.76 |  |
| 30q of helpful RT | 0.74 | 1.04 | 0.37 | TRUE | 26.60 | 0.83 |  |
| 50q of helpful RT | 1.28 | 1.38 | 0.47 | TRUE | 41.01 | 0.20 |  |
| 70q of helpful RT | 1.77 | 1.79 | 0.60 | TRUE | 47.38 | 0.02 |  |
| 90q of helpful RT | 2.32 | 2.43 | 0.81 | TRUE | 45.64 | 0.14 | |
|  |  |  |  |  |  |  | |
| **Control group: Other** |  |  |  |  |  |  | |
| % of harmful decision | 0.59 | 0.59 | 0.27 | TRUE | 45.64 | 0.02 | |
| Mean harmful RT | 1.85 | 2.07 | 0.53 | TRUE | 35.27 | 0.42 | |
| 10q of harmful RT | 0.92 | 1.24 | 0.41 | TRUE | 17.26 | 0.78 | |
| 30q of harmful RT | 0.84 | 1.02 | 0.37 | TRUE | 32.44 | 0.49 | |
| 50q of harmful RT | 1.30 | 1.32 | 0.43 | TRUE | 47.89 | 0.04 | |
| 70q of harmful RT | 1.65 | 1.68 | 0.50 | TRUE | 49.56 | 0.06 | |
| 90q of harmful RT | 2.19 | 2.26 | 0.63 | TRUE | 48.55 | 0.11 | |
|  |  |  |  |  |  |  | |
| Mean helpful RT | 1.85 | 2.18 | 0.64 | TRUE | 67.16 | 0.51 | |
| 10q of helpful RT | 1.01 | 1.31 | 0.49 | TRUE | 26.97 | 0.61 | |
| 30q of helpful RT | 0.69 | 1.05 | 0.39 | TRUE | 18.88 | 0.92 | |
| 50q of helpful RT | 1.20 | 1.37 | 0.48 | TRUE | 37.36 | 0.36 | |
| 70q of helpful RT | 1.69 | 1.77 | 0.60 | TRUE | 44.69 | 0.14 | |
| 90q of helpful RT | 2.26 | 2.39 | 0.77 | TRUE | 44.49 | 0.18 | |

*Notes: 10q ~ 90q: 10^th^ ~ 90^th^ quantile of RT distribution; Credible: whether observed data falls in the 95% credible interval of the simulated data; Mahalanobis: Mahalanobis distance of the observed data from the center of distribution of the simulated data.*
